# Biomaterial-assisted tumor therapy: A brief review of hydroxyapatite nanoparticles and its composites used in bone tumors therapy

**DOI:** 10.3389/fbioe.2023.1167474

**Published:** 2023-04-07

**Authors:** Quan Zhang, Lei Qiang, Yihao Liu, Minjie Fan, Xinxin Si, Pengfei Zheng

**Affiliations:** ^1^ Department of Orthopaedic Surgery, Children’s Hospital of Nanjing Medical University, Nanjing, China; ^2^ Jiangsu Key Laboratory of Marine Bioresources and Environment, Co-Innovation Center of Jiangsu Marine Bio-industry Technology, Jiangsu Key Laboratory of Marine Pharmaceutical Compound Screening, Jiangsu Ocean University, Lianyungang, China; ^3^ School of Materials Science and Engineering, Southwest Jiaotong University, Chengdu, China; ^4^ Shanghai Key Laboratory of Orthopedic Implant, Department of Orthopedic Surgery, Shanghai Ninth People’s Hospital, Shanghai Jiao Tong University School of Medicine, Shanghai, China

**Keywords:** hydroxyapatite nanoparticles, tumor cell apoptosis, bone tumor therapy, drug delivery, PTT, MFH

## Abstract

Malignant bone tumors can inflict significant damage to affected bones, leaving patients to contend with issues like residual tumor cells, bone defects, and bacterial infections post-surgery. However, hydroxyapatite nanoparticles (nHAp), the principal inorganic constituent of natural bone, possess numerous advantages such as high biocompatibility, bone conduction ability, and a large surface area. Moreover, nHAp’s nanoscale particle size enables it to impede the growth of various tumor cells *via* diverse pathways. This article presents a comprehensive review of relevant literature spanning the past 2 decades concerning nHAp and bone tumors. The primary goal is to explore the mechanisms responsible for nHAp’s ability to hinder tumor initiation and progression, as well as to investigate the potential of integrating other drugs and components for bone tumor diagnosis and treatment. Lastly, the article discusses future prospects for the development of hydroxyapatite materials as a promising modality for tumor therapy.

## Graphical Abstract

**Figure:**
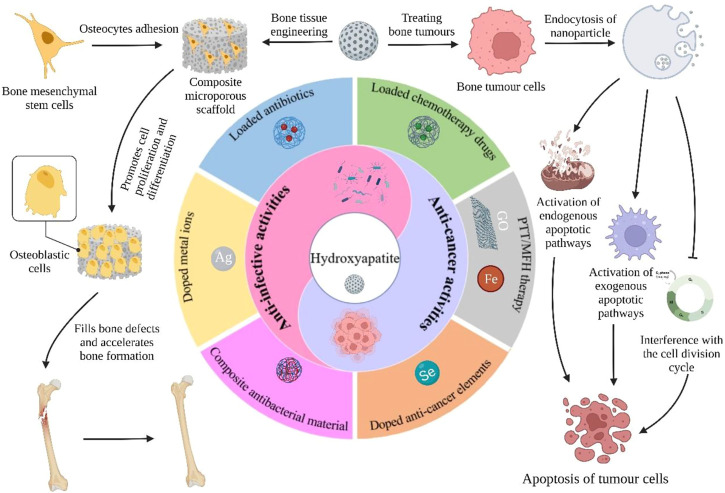

## 1 Introduction

The World Health Organization classifies bone tumors as “benign,” “intermediate” (locally aggressive or occasionally metastatic), or “malignant” (Cortini et al., 2019). Osteoid osteoma is a common benign tumor that affects children and adolescents, while giant-cell tumors are intermediate and locally aggressive. The most common primary malignant tumor of bone is osteosarcoma, which occurs mainly in children and adolescents and affects the distal femur, proximal tibia, and humerus. Unfortunately, fewer than 30% of patients with osteosarcoma who experience metastasis or recurrence are alive 5 years after diagnosis (Isakoff et al., 2015a; Belayneh et al., 2021). Malignant bone tumors are known for their rapid growth, aggressiveness, and potential for metastasis, and often result in severe pain, pathologic fractures, hypercalcemia, cachexia, as well as damage to surrounding tissue (Bădilă et al., 2021).

The standard treatment for both benign and malignant bone tumors involves aggressive surgical removal, coupled with adjunctive therapies such as radiotherapy, chemotherapy, targeted therapy, or biologic agents (Isakoff et al., 2015a). The typical chemotherapeutic drugs used to treat osteosarcoma include adriamycin (DOX), methotrexate (MTX), cisplatin (DDP), and isocyclophosphamide (IFO) (Bădilă et al., 2021). However, traditional chemotherapy agents are hindered by several limitations, including poor aqueous solubility, short residence time in the bloodstream, inadequate targeting, and delayed controlled release. Furthermore, patients may develop multidrug resistance, which can increase the efflux of chemotherapy drugs and weaken their antitumor effects (Zhang et al., 2018; Maleki Dana et al., 2021). While resection can alleviate clinical symptoms and enhance neurological function, the removal of significant portions of bone tissue and the persistence of residual tumor cells may result in the recurrence and metastasis of bone tumors (Maleki Dana et al., 2021). Alongside adjuvant therapy, bone repair at the defect site is necessary to alleviate the patient’s physical burden.

The standard procedure for treating bone defects involves the use of autologous bone and allogeneic bone grafts. An autologous bone graft implant uses tissue derived from the patient and is considered the ideal source of bone for internal fixation, as it exhibits exceptional histocompatibility, osteoconductivity, and non-immunogenic properties (Brydone et al., 2010; Gibbs et al., 2014). Allografts are grafts obtained from cadavers or donors and are beneficial for osteoinduction due to their high content of bone morphogenetic proteins and other growth factors. These factors promote the formation of bone progenitor cells *via* the differentiation of various types of stem cells, particularly mesenchymal stem cells (Amini et al., 2012; Bharadwaz and Jayasuriya, 2020). Bone grafting is a common procedure for the clinical repair of bone defects. However, autologous and allogeneic transplants have several drawbacks, including heightened surgical risks, local tissue damage at the donor site, restricted bone volume, postoperative infections, immune system rejection, and disease transmission (Baroli, 2009; Dimitriou et al., 2011; Mishra et al., 2016). As a result, substantial research has been conducted to identify alternative materials for bone filling. This research emphasizes bioactive materials that maintain the integrity and functionality of bone while stimulating bone growth. In the early stages, implants provide robust mechanical support to the defect site. In the medium term, implants furnish a three-dimensional (3D) space and a porous, interoperative matrix for newly grown bone tissue. The matrix can absorb bioactive factors or “seed” cells and supply nutrients and waste transportation, expediting tissue repair and vascularization. In the long run, the newly generated bone tissue contributes to bodily activities (Amini et al., 2012; Guo and Ma, 2014; Bharadwaz and Jayasuriya, 2020).

Calcium phosphate-based materials are widely recognized as the most effective materials for supporting the repair, replacement, and regeneration of bone. These materials were initially used for bone repair in 1920 and have been extensively studied since the 1950s for medical implant applications. Hydroxyapatite (HAp) was first employed as an implant material in 1971 (Monroe et al., 1971; Fox et al., 2012).

Natural bone is a unique porous composite material comprising organic components (primarily collagen, which makes up 90%–95% of its composition) and inorganic components (mainly hydroxyapatite, Ca_10_(PO_4_)_6_(OH)_2_, a nano-crystalline form of calcium phosphate material). Although the nano-sized hydroxyapatite particles (20–40 nm) provide excellent mechanical strength, they are relatively weak when it comes to torsional resistance. Conversely, the organic component in the form of collagen offers high toughness (Wubneh et al., 2018). HAp is considered to be the most suitable substitute material for repairing human bone tissue because it is the primary inorganic component of human hard tissue and is highly conducive to close interaction with cells and tissues in the biological environment.

Hydroxyapatite materials have shown great potential as implants due to their ability to directly connect with normal bone tissue without forming fibrous tissue interfaces. These materials possess good plasticity, osteoconduction, non-toxicity, and *in-situ* biodegradability, and can transport active ions. However, their limited load-bearing capacity, poor shear and bending abilities, and brittleness pose challenges for their application. Additionally, their degradation time may be too long to match the bone regeneration rate (Motskin et al., 2009; Mann et al., 2011; Zhou and Lee, 2011). Pure hydroxyapatite also lacks the ability to recruit cells, regulate immunity, or promote blood vessel regeneration. To overcome these limitations, researchers have explored mixing hydroxyapatite with other materials such as biopolymers and metal ions, which can enhance the physical and biological properties of the resulting composites. This approach has improved the mechanical strength and stability of the materials, as well as bone cell adhesion, proliferation, and differentiation. Moreover, it has expanded the range of applications and enabled the composites to acquire new functions (Shuai et al., 2021; Feng et al., 2022). Recent studies have focused on different preparation methods and clinical applications of hydroxyapatite-based materials as bone fillers, scaffold materials, implant coatings, and drug carriers (Szcześ et al., 2017).

Recent research on hydroxyapatite nanoparticles (nHAp) has yielded some notable findings. In terms of physical and chemical properties, nanoscale hydroxyapatite particles exhibit several advantages over ordinary HAp particles, including increased aspect ratio and total pore volume, greater surface area, and improved dispersibility. These advantages enhance material density, improve fracture toughness, and reduce biodegradability (Iyyappan et al., 2016; Kubasiewicz-Ross et al., 2017; Shahrezaie et al., 2018). In terms of biological activity and biocompatibility, studies have shown that nHAp with diameters of approximately 20 nm can promote cell growth and inhibit cell apoptosis more effectively than nHAp with diameters of 80nm, according to Shi et al. (2009). Additionally, Zhang K et al. observed new bone formation at the pore walls of nHAp scaffolds in rabbit femurs, and found that the serum alkaline phosphatase concentration in the nHAp scaffold group was significantly higher than that in the control or normal group, indicating the excellent bone induction ability of nHAp (Zhang et al., 2019). By mimicking the nano-sized hydroxyapatite particles dispersed between collagen fibers in natural bone, materials based on nHAp as the basic component can closely resemble natural bone.

In recent years, numerous studies have demonstrated that nHAp can inhibit the proliferation of various cancer cells, including osteosarcoma cells, while inducing apoptosis, without affecting normal cells. This mechanism has been demonstrated in multiple ways, making nHAp composite materials a hot topic in bone tumor prognosis materials (Dey et al., 2014; Jin et al., 2014; Kamitakahara et al., 2016). Many new functional nHAp composite materials have been shown to possess anti-tumor abilities and can effectively address postoperative bone defects, tumor recurrence, and metastasis. As carriers, these materials can locally release drugs, eliminate residual tumor cells, and avoid severe systemic toxicity. Additionally, nHAp composite materials with excellent photothermal or magnetic heating properties can serve as photothermal or magnetic agents, enabling localized effects to effectively ablate tumor cells.

This article presents a comprehensive overview of the application of nHAp in the treatment of bone tumors. Firstly, the principle and mechanism of nHAp’s anti-tumor effect are thoroughly analyzed and summarized. Secondly, representative studies of nHAp derivatives in the treatment of bone tumors are reviewed and classified based on their specific roles in the treatment process, highlighting the research hotspots and corresponding achievements in this field. The article emphasizes the significance of incorporating other substances to modify the material’s properties. Ultimately, this article aims to provide preliminary information that can assist in designing and planning superior-performing nHAp materials for the treatment of bone tumors.

## 2 The anti-tumor mechanism of nHAp

Compared to larger-sized HAp materials, nHAp exhibits superior physicochemical properties and biological activity, making it increasingly attractive in the field of bone tissue engineering. Notably, several studies have reported that nHAp has inherent anti-tumor effects on various cancer cell lines and animal cancer models. These effects appear to be mediated through several mechanisms, including the reduction of tumor cell adhesion, the induction of apoptosis and cell cycle arrest, and the downregulation of telomerase activity. By summarizing these mechanisms, we can gain a better understanding of nHAp’s anti-tumor abilities and establish a theoretical foundation for its potential use in the treatment of bone tumors.

### 2.1 nHAp induces tumor cell apoptosis

**FIGURE 1:**
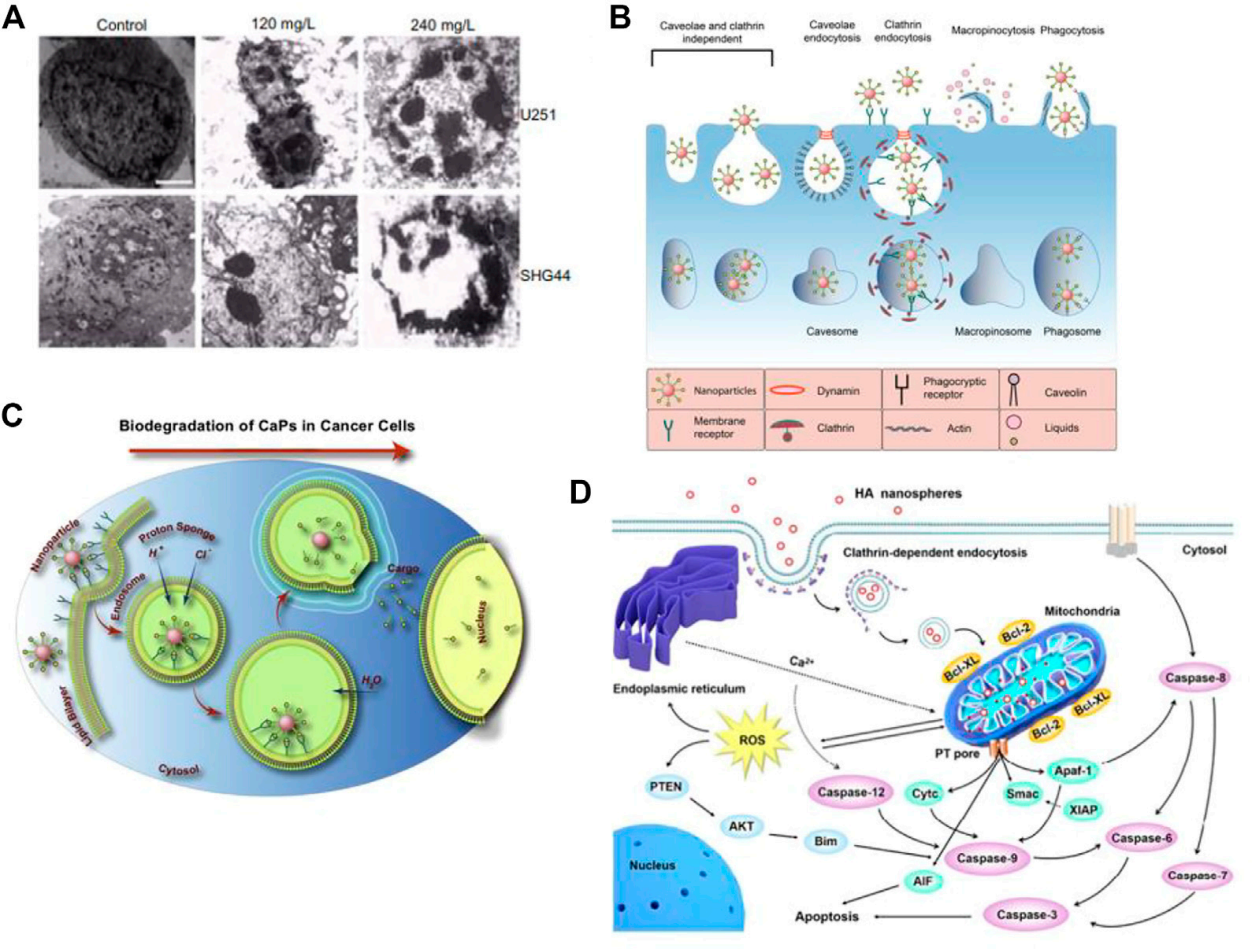
Cytotoxicity of nHAp on tumor cells. **(A)** Treatment with nHAp caused changes in the morphology of human glioma SHG44 cells and U251 cells. **(B)** Tumor cells engulf nHAp through various endocytic mechanisms. **(C)** nHAp is degraded in the acidic microenvironment of lysosomes, and H^+^ diffuses into the cytoplasm through proton pumps. To maintain charge neutrality, Cl^−^ and H_2_O enter the lysosome, causing lysosome swelling and rupture, releasing large amounts of Ca^2+^, PO_4_^3-^, and OH^−^, reaching toxic concentrations, inducing cell apoptosis. **(D)** Illustration of the possible molecular mechanism of the inhibitory effect of nHAp on 4T1 cells. **(A)** Reproduced with permission (Chu et al., 2012). Copyright 2012, Int J Nanomedicine. **(B)**, **(C)** Reproduced with permission (Khalifehzadeh and Arami, 2020). Copyright 2020, Advances in Colloid and Interface Science. **(D)** Reproduced with permission (Zhao et al., 2018). Copyright 2017, ACS Nano.

Cell apoptosis is a programmed and orderly cell death process regulated by genes to maintain internal environmental stability. In response to physiological or pathological stimuli, cells and tissues undergo apoptosis, which differs significantly from necrosis. Wu et al. (2019) conducted an experiment in which they injected nHAp and melanoma cell suspension into nude mice to observe the development of tumor tissue and determine the ability of nHAp to inhibit tumor growth. The results showed that the tumor tissue volume in the nHAp group was consistently smaller than that in the control group at each experimental recording point. Similarly, Chu et al. (2012) verified the inhibitory effect of nHAp on human glioma cells U251 and SHG44. Figure 1A illustrates significant changes in tumor cell morphology, such as cell volume shrinkage, chromatin condensation, and fragmentation, which are typical characteristics of apoptosis after treatment with 120 mg/L and 240 mg/L nHAp for 48 h. Additionally, the growth of tumor xenografts in nude mice treated with nHAp was significantly inhibited. Numerous studies have demonstrated that nHAp can induce apoptosis in various cancer cell lines (Yuan et al., 2010; Xu Z. et al., 2012; Jin et al., 2014; Cui et al., 2016; Zhang et al., 2019).

#### 2.1.1 Calcium homeostasis imbalance causes apoptosis

Maintaining calcium homeostasis is essential for various metabolic activities in cells. An imbalance in calcium homeostasis, characterized by an uncontrolled increase in calcium ion concentration in the cytoplasm, can cause cell apoptosis or even autophagy (Anand et al., 2019). As illustrated in Figure 1B, tumor cells can uptake nHAp through passive transport, active transport, and various endocytosis processes. Due to the higher metabolic rate of tumor cells, more nHAp is internalized into the cytoplasm of tumor cells compared to normal cells, and the cytotoxicity of nHAp to tumor cells may be attributed to this mechanism (Marycz et al., 2016). Studies have found that nHAp induces a sustained increase in intracellular Ca^2+^ in tumor cells, but only a transient increase in normal control cells (Sun et al., 2016; Khalifehzadeh and Arami, 2020). This may be due to the different metabolic pathways of tumor cells compared to normal cells. Specifically, tumor cells tend to metabolize more glucose *via* glycolysis to generate pyruvate, which is then converted to lactate, leading to a lower pH in the tumor cell microenvironment (pH 5.0–5.5) than in normal cells (Counihan et al., 2018). As shown in Figure 1B, when nHAp is internalized by tumor cells, it quickly degrades in the low pH environment of the cytoplasm and lysosomes, releasing a large amount of Ca^2+^, PO_4_^3-^, and OH^−^(Khalifehzadeh and Arami, 2020). These ions diffuse into the cytoplasm and reach toxic concentrations, disrupting calcium ion homeostasis, and ultimately leading to tumor cell apoptosis.

After treatment with nHAp, there was a significant upregulation of adenosine triphosphate calcium phosphatase-1 (ATP2A1) and sodium-dependent phosphate transporter protein 1 (SLC20A1) gene expression in the sarcoplasmic reticulum/endoplasmic reticulum, both of which are involved in cytosolic Ca^2+^ transport in tumor cells. In contrast, the expression of calcitonin-1 (STC1) and voltage-dependent calcium channel subunit-2/Δ-1 (CACNA2D1) was downregulated (Zhu et al., 2017b). In the plasma membrane, the expression of several genes related to Ca^2+^ transport, such as ATP2B2, SLC8A1, TRDN, SRL, CACNB1, RYR1, CASQ2, CALB1, STC1, and CACNA2D1, was also found to differ following nHAp treatment, as reported in a recent study (Zhang et al., 2019). These findings suggest that nHAp may alter the permeability and retention of tumor cell membranes, resulting in excessive uptake of Ca^2+^ and dysregulation of intracellular Ca^2+^ homeostasis.

#### 2.1.2 Activation of mitochondria-mediated endogenous apoptosis

After comparing the effects of nHAp on lung cancer cells (A549) and normal bronchial epithelial cells (16 HBE), it was discovered that nHAp significantly increased the expression of the pro-apoptotic BAX protein in A549 cells while reducing the expression of the anti-apoptotic Bcl-2 protein (Xue et al., 2017). The BAX protein plays a critical role in the mitochondrial apoptosis pathway, while the Bcl-2 protein is responsible for regulating the permeability of the mitochondrial membrane. By altering the ratio of pro-apoptotic to anti-apoptotic proteins, nHAp exacerbated the permeability of the mitochondrial membrane, resulting in the release of apoptotic factors such as cytochrome C (Cyt C), Smac protein, APAF-1, and AIF into the cytoplasm. Cyt C plays a crucial role as an electron transfer agent in biological oxidation, and its accumulation in the cytoplasm can block downstream electron transfer and generate reactive oxygen species (ROS) (Xu et al., 2012; Zhao et al., 2018). Furthermore, the expression of the antioxidant factors glutathione (GSH) and CAT decreased, leading to an accumulation of excess ROS in the cytoplasm. This, in turn, inhibited the tumor suppressor gene PTEN. PTEN regulates the PI3K/AKT pathway in cells, which is responsible for inhibiting cell proliferation and migration. The binding of Smac protein and X-linked inhibitor of apoptosis protein (XIAP) triggers an interaction between caspases, leading to the activation of caspase-8 and caspase-9 *via* self-cleavage. This activation triggers a cascade reaction of caspases, ultimately activating downstream effector proteins caspase-3, caspase-6, and caspase-7. This process induces the mitochondrial-mediated intrinsic apoptosis pathway, ultimately resulting in the death of tumor cells. For instance, Chen et al. (2007) conducted a study where they treated human gastric cancer SGC-7901 cells with 500 μg/mL of nHAp. The results showed a significant 2.7-fold increase in the activity of caspase-3 and a 1.9-fold increase in the activity of caspase-9, suggesting that nHAp can trigger the activation of caspase-3 and caspase-9 in tumor cells. Excessive Ca^2+^ in the cytoplasm may also be a factor that triggers mitochondrial dysfunction in the intrinsic apoptosis pathway (Zhang et al., 2019). As illustrated in Figure 1D, nHAp disrupts calcium homeostasis in tumor cells through endocytosis, leading to the accumulation of reactive oxygen species (ROS) in the cells. Ultimately, this causes an increase in oxidative stress through a negative feedback system, activating the mitochondrial-mediated apoptotic pathway and the phosphatidylinositol-3-kinase/protein kinase B pathway (Xu et al., 2012; Zhao et al., 2018).

In the group treated with nHAp, a decrease in the expression of the Ki-67 protein, a marker of cell proliferation, and an increase in TUNEL (terminal deoxynucleotidyl transferase-mediated deoxyuridine triphosphate nick end labeling) expression were observed (Zhang et al., 2019). The observed phenomena indicate a lower proliferative ability of tumor cells and a greater induction of apoptosis following treatment with nHAp. Additionally, we noted chromatin aggregation at the nuclear edge, expansion of the nuclear membrane, mitochondrial swelling and vacuolization, as well as the formation of apoptotic vesicles in tumor cells of the nHAp-treated group (Zhang et al., 2019). These results suggest that nHAp may trigger the mitochondria-mediated apoptotic pathway in tumor cells *in vivo*.

#### 2.1.3 Activation of extrinsic apoptotic pathway

The histology of tumors treated with nHAp revealed multiple types of immune cells, particularly M1 macrophages, at the interface between the muscle and the tumor (Zhang et al., 2019). In the nHAp group, the expression of genes expressed by different types of immune cells was significantly upregulated, including tumor necrosis factor (TNF), DCSTAMP, MRC1, CLEC7A, TLR4, RLA-DR-Alpha, and GZMK. These findings suggest that nHAp may stimulate the innate immune system, recruit several types of immune cells (such as macrophages, dendritic cells, monocytes, and cytotoxic lymphocytes) into the tumor microenvironment, and activate the death receptor-mediated exogenous apoptotic pathway. This activation occurs by triggering the intracellular apoptotic enzyme caspase through extracellular signaling (Huang et al., 2013).

Macrophages can change their phenotype in response to changes in the microenvironment by undergoing polarization, with the main phenotypes being classically activated macrophages (M1 type) and selectively activated macrophages (M2 type). nHAp was found to activate the polarization of M1 macrophages *in vivo*, enhancing their ability to phagocytose and kill pathogens, upregulate the expression of proinflammatory cytokines, and show cytotoxic effects on tumor growth. ELISAs demonstrated that co-culture of macrophages with nHAp contributed to the secretion of proinflammatory cytokines and significantly enhanced expression of TNF-α and monocyte chemotactic protein-1 while reducing expression of insulin-like growth factor and vascular endothelial growth factor C. This reduction could cause hemorrhage, apoptosis, and necrosis in tumor tissue (Zhao et al., 2018; Zhang et al., 2019).

### 2.2 Interference with the tumour cell division cycle

Cell proliferation occurs in different phases of the cell cycle, with G1 and S phases responsible for preparing the main substances required for division, such as the synthesis of key proteins and DNA replication. Progression of the cell cycle is dependent on various environmental conditions and can be significantly altered by treatment with exogenous substances (Benedetti et al., 2015). In tumor cells, the endocytosis process is different from that of normal cells, allowing for the distribution of many nHAp particles around the endoplasmic reticulum of tumor cells (Furmanik and Shanahan, 2018). Due to its high adsorption capacity to ribosomes, nHAp reduces the binding of messenger-RNA to appropriate sites in ribosomes, thus inhibiting the synthesis of key proteins and affecting DNA replication in tumor cells. After treatment with nHAp, the percentage of tumor cells in different stages of the cell cycle showed significant changes. The number of cells in the G0/G1 phase increased by 24.2%, while the number of cells in the S phase and G2/M phase decreased by 38.2% and 12.9%, respectively. This indicates that nHAp can directly interfere with protein transcription as an exogenous substance, leading to the lack of key proteins necessary for tumor cell division, resulting in cell cycle arrest in the G0/G1 phase and further inducing inhibition of tumor cell proliferation (Han et al., 2014; Zhang et al., 2019; Xue et al., 2020).

### 2.3 Inhibits telomerase activity

The development of tumors is closely linked to telomerase activity, which helps to maintain the length of telomeres and chromosome stability. When telomerase activity is downregulated, telomeres can shorten, leading to cell senescence and eventually, cell death (Zeng et al., 2018; McKelvey et al., 2020). Research has demonstrated that nHAp can reduce telomerase levels in tumor cells (Motskin et al., 2009). Treatment of tumor cells with nHAp at varying concentrations has shown that telomerase activity decreases as nHAp concentration increases, while the apoptosis rate increases with higher nHAp content (He et al., 2022).

### 2.4 Inhibits tumor cell adhesion and migration

The surface of nHAp is highly positively charged, which allows it to easily adhere to the cell membrane and activate endocytosis. Yin MZ et al. conducted an *in vitro* study on the interaction between Bel-7402 liver cancer cells and nHAp. The study found that, compared to the untreated group, the Bel-7402 cell suspension containing nHAp remained uniformly distributed in the medium after 24 h without aggregation or cell adhesion (Yin et al., 2006). These results suggest that nHAp can affect the adhesion of tumor cells. The possible reason for this interaction is that the level of glycosylation on the surface of tumor cells, such as sialic acid structures, increases, resulting in an increase in negative charge on the cell membrane surface. This makes positively charged nHAp more likely to adhere to the tumor cell membrane. When tumor cells are coated with nHAp, the function of their cell membrane is affected, inhibiting their aggregation and adhesion to each other and ultimately inhibiting their proliferation (Han et al., 2014; Ionescu et al., 2020).

In another study, Wang R et al. investigated the intrinsic mechanism by which nHAp inhibits tumor cell growth, migration, and invasion (Wang et al., 2021). The study found that nHAp significantly reduced the cell viability, migration, and invasion ability of osteosarcoma cells OS-732 in a concentration-dependent manner from 0 μg/ml to 800 μg/ml. In a BALB/c nude mouse tumor model, the tumor weight in the blank group was 6.1 times higher than that in the group treated with 400 μg/ml of nHAp, demonstrating that nHAp can effectively inhibit *in vivo* tumor growth. RNA-seq analysis revealed that nHAp treatment downregulated the activity of the FAK/PI3K/Akt signaling pathway, regulating the growth and development of OS-732 cells. These results provide further evidence for the effectiveness of nHAp in treating osteosarcoma.

To summarize, nHAp has demonstrated inhibitory effects on various cancer cell lines, although these effects can vary depending on the cell type and cannot be uniformly evaluated. Tang W et al. studied the cytotoxicity of rod-shaped nHAp on three types of human cancer cells (gastric cancer cells [MGC5-2], cervical adenocarcinoma cells [HeLa], and liver cancer cells [HepG2]) and normal human liver cells ([L-02]) (Tang et al., 2014). The results showed that nHAp significantly inhibited cell proliferation and induced apoptosis in cancer cells, with the strength being in the order of MGC80-3 > HepG2 > HeLa. However, nHAp had no effect on normal liver cells (L-02), indicating that the uptake and internalization activity of nHAp in tumor cells is much higher than that in normal cells, leading to a difference in the degree of proliferation inhibition between normal and tumor cells, and low cytotoxicity is displayed in normal cells (Han et al., 2014).

Moreover, changes in the physicochemical properties of nHAp, such as particle size, shape, and dosage, can result in different anti-tumor effects against the same type of cancer cell line. In a study by Yuan et al. (2010), the effect of nHAp particle size on its anti-tumor activity against human liver cancer HepG2 cells was investigated. nHAp particles of varying sizes, ranging from 20 to 180 nm, were synthesized using the sol-gel method. The results showed that the anti-tumor activity against HepG2 cells was dependent on the particle size of nHAp, with the most effective size being 45 nm, followed by 26 nm, 78 nm, and 175 nm. Interestingly, the smallest nHAp did not exhibit the most significant toxicity despite having the highest specific surface area. Additionally, the shape of nHAp is another important factor that affects its anti-tumor activity. In a study by Xu et al. (2012), four types of nHAp (short rod-like, long rod-like, spherical, and needle-like) were compared. Needle-like and short rod-like nHAp induced more significant cell damage, with increased apoptosis rate and Cyt C expression, compared to spherical and long rod-like nHAp. This suggests that changes in the aspect ratio between different shapes could lead to a change in the specific surface area of nHAp, which could more effectively interact with the tumor cell membrane or organelles. Numerous studies have demonstrated that nHAp inhibits tumor cell proliferation in a concentration-dependent manner, and its effectiveness is closely linked to the type of cell death induced (Xu et al., 2012; Martin and Henry, 2013; Han et al., 2014). For instance, when treating human glioma U251 and SHG44 cells with a low dose of short rod-like nHAp (120 mg/L) for 48 h, typical apoptotic changes occurred, whereas a high dose (240 mg/L) of the same type of nHAp caused necrotic changes such as cell swelling, membrane rupture, and cell content leakage (Chu et al., 2012). Due to its high biocompatibility, bone-inducing properties, and low cytotoxicity to normal cells, nHAp is a promising anti-tumor agent for the treatment of bone tumors. Furthermore, choosing the appropriate concentration and shape of nHAp can lead to safer and more effective treatments for various types of tumors.

## 3 Application of nHAp in the treatment of bone tumors

For patients with primary malignant bone tumors or other metastatic tumors, surgical removal of the lesion is often not enough to eliminate all tumor cells. Tumor recurrence and bone defects following surgery are among the main reasons for the low survival rate and poor quality of life for bone tumor patients (Luetke et al., 2014). Moreover, bone tumors are often unresponsive to chemotherapy and tend to develop drug resistance (He et al., 2014; Luetke et al., 2014). However, with the emergence of bone tissue engineering, various innovative therapies have been developed for the treatment of bone tumors. These include drug delivery systems (DDS), photothermal therapy (PTT), magnetic hyperthermia (MFH), and other therapies. Compared to traditional therapies, most innovative therapies offer multiple benefits, such as lower toxicity, enhanced functionality, and reduced physical burden for the patient. nHAp plays a key role in many of these therapies.

### 3.1 Application of nHAp in antitumor drug delivery system

To prevent the recurrence and spread of residual tumor cells after surgery, further chemotherapy or radiotherapy is often necessary. However, these treatments can have serious toxic side effects on vital organs such as the liver and kidneys. To address these issues, implanting drug delivery systems (DDS) has become a viable strategy. DDS can release drugs directly to the target tissue for an extended period, reduce the drug dosage, maintain the effective drug concentration, and minimize the risk of non-selective and highly toxic systemic drug toxicity (Sun et al., 2018). Several studies have utilized nHAp as an anti-tumor drug delivery system, which are summarized in Table 1.

**TABLE 1:** The role of nHAp in antitumor drug delivery systems.

Material	Method of synthesis	Tumor therapy method	Effect	Ref
nHAp, BSA, PTX	Coprecipitation	Chemotherapy	This material inhibits the proliferation of osteosarcoma cells (143B) and reduces their invasion ability. Additionally, it induces and upregulates the expression of osteogenic genes, promotes calcium ion mineralization and deposition, and repairs bone defects	Liu et al. (2021)
nHAp, FA, DOX	*In situ* coprecipitation; Hydrothermal	Chemotherapy	By utilizing the specific combination of FA and FR for targeted delivery of DOX, the anti-tumor efficiency is improved, while systemic side effects are reduced	Sun et al. (2018)
nHAp, PLGA, ADM	Sol–gel	Chemotherapy	Local sustained-release chemotherapeutic drugs exhibit strong anti-tumor activity against osteosarcoma MG63 cells and promote bone repair	Rong et al. (2016)
nHAp、CS、Zol	*In situ* precipitation	Chemotherapy	This material upregulates pro-apoptosis-related genes, inhibits the proliferation of giant cell tumor cells of bone (GCTBs), prevents infection in damaged areas, and accelerates bone defect repair	Lu et al. (2018b)
nHAp, PLLA, MET	Selective laser sintering	Chemotherapy	This material induces osteosarcoma cell Saos-2 death and promotes the osteogenic differentiation of hBMSCs	Tan et al. (2021)
nHAp, Se, CC	Coprecipitation	Chemotherapy	The modification of catechin activates the caspase-3 pathway by generating reactive oxygen species (ROS), which induces apoptosis of the human osteosarcoma MNNG/HOS cell line. This modification also enhances the anti-tumor activity of the material and reduces the toxic effect of selenium on normal cells	Khan et al. (2019)
nHAp, Co^2+^, Fe^3+^, FU	Microwave assisted wet precipitation	synergistic chemo-hyperthermia therapy	This material exhibits temperature-controlled drug release behavior, increases the release percentage of FU, has obvious proliferative activity on the mouse fibroblast cell line L929, and inhibits the growth of the human osteosarcoma cell line MG63	Sangeetha et al. (2019)
nHAp, GO, HSV-TK, GCV	Hydrothermal	Gene therapy	The addition of GO increases the specific surface area of nHAp, providing a large number of binding sites for HSV-TK. GO also serves as a new type of efficient gene carrier for tumor therapy, delivering a “suicide” gene into human breast cancer cell lines MDA-MB-231 and MCF7. Upon administration of GCV, which induces tumor cell apoptosis in response to DNA damage, the system exhibits a significant antitumor effect	Cheang et al. (2018)
nHAp, PEI, siRNA	Hydrothermal	Gene therapy	The positive surface potential of nHAp can improve the stability and transfection efficiency of the system. Furthermore, it can improve the cell uptake efficiency and reduce the cytotoxicity to the human liver cancer cell line BEL-7402	Xu et al. (2015)
FHAPS, DOX, siRNA	Gas foaming	Chemotherapy;Gene therapy	The material has a rough surface, and the particle size and shape are controllable. It can be loaded with DOX (9.1%) and siRNA (2.0%). The DOX can be effectively delivered to the breast cancer cell MCF-7/ADR to exert its toxic effect, while the siRNA can be transfected to interfere with the expression of target proteins in cells	Liu et al. (2020b)
LHAp, PLGA, DOX	Electrospinning	Chemotherapy	Compared with the DOX@nHAp/PLGA scaffold made of needle-like nHAp, DOX@LHAp/PLGA is less toxic to HepG2 cells. However, it greatly alleviates the burst release of DOX and maintains a long-term controlled release effect	Luo et al. (2019)

Abbreviations: ADM, doxorubicin hydrochloride; BSA, bovine serum albumin; CC, catechins; CPT, cisplatin; CS, chitosan; DOX, docetaxel; FA, folic acid; FHAPS, flower-like HAp, spheres; FR, folate receptor; FU5, fluorouracil; GCV, ganciclovir; GO, graphene oxide; HSV-TK, herpes simplex virus thymidine kinase gene; LHAp, layered nanohydroxyapatite; MET, metformin; PEI, polyethylenimine; PLGA, poly (lactic-co-glycolic acid); PLLA:poly-L-lactic acid; Se, selenium; PTX, paclitaxel; siRNA, small interfering RNA; zol, zoledronic acid.

**FIGURE 2:**
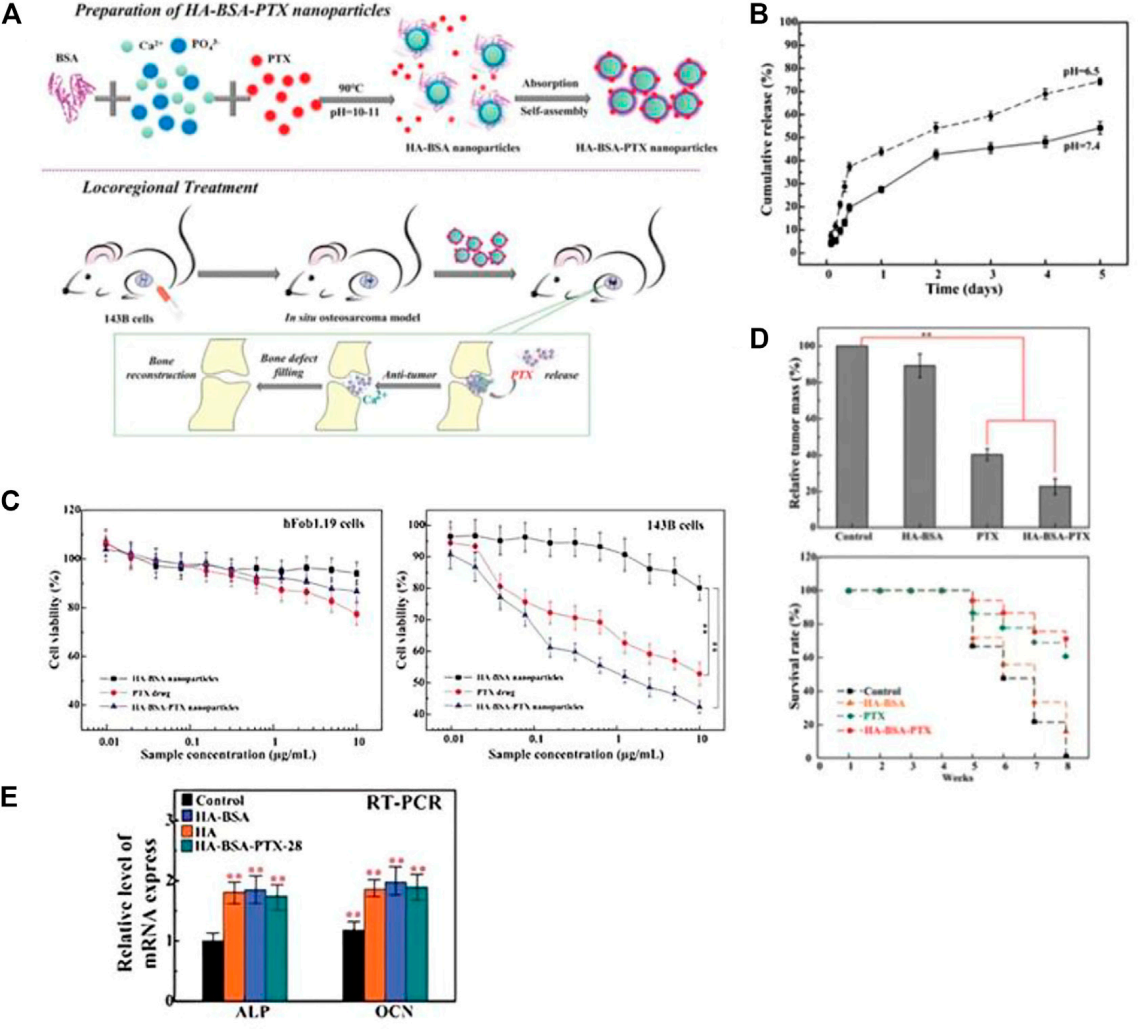
**(A)** Schematic diagram of the preparation process of HA-BSA-PTX nanoparticles and the experimental design of the *in vivo* osteosarcoma model. **(B)** *In vitro* drug release evaluation of HA-BSA-PTX nanoparticles in PBS solution at pH 7.4 and 6.5 with Tween 80 (0.1%, v/w). **(C)** MTT assay was used to evaluate the *in vitro* toxicity of HA-BSA nanoparticles, free PTX, and HA-BSA-PTX nanoparticles on hFob1.19 cells and 143B cells. With increasing dosage, HA-BSA-PTX nanoparticles showed the best toxicity to 143B cells and lower toxicity to hFob1.19 cells than PTX alone. **(D)** Weight of osteosarcoma tissue treated with control (PBS), HA-BSA nanoparticles, PTX alone, and HA-BSA-PTX nanoparticles, and survival time of nude mice. **(E)** mRNA expression levels of ALP and OCN after osteogenic induction for 14 days with co-culture of BMSCs cells and each group at a concentration of 10 μg/mL. Reproduced with permission (Liu et al., 2021). Copyright 2020, Advanced Healthcare Materials.

In Figure 2A, Liu Y et al. utilized BSA as a model protein to encapsulate and load PTX, which was then combined with rod-shaped nHAp for localized treatment of osteosarcoma. The resulting drug delivery system, designated as HA-BSA-PTX, had a diameter of approximately 55 nm and a drug loading efficiency of 32.17 wt% (Liu et al., 2021). Figure 2B depicts the release curve of HA-BSA-PTX in a PBS solution at pH 6.5, which showed no significant burst release of PTX in the short term but sustained release for 5 days and a higher release rate compared to the PBS solution at pH 7.4. These findings suggest that BSA, as a drug carrier, improved the hydrophilicity of PTX and exhibited excellent sustained release properties. Additionally, BSA enhanced the release of PTX in the low pH microenvironment of the tumor site, thereby reducing the toxicity of the drug to normal cells. The composite material’s potential for *in situ* treatment of osteosarcoma after surgery was further evaluated through *in vitro* cell experiments and *in vivo* animal experiments. The study revealed that the drug-loaded nanoparticles had a dual function of reducing the cytotoxicity to human fetal osteoblast cells (hFOB 1.19) and significantly inhibiting the proliferation and invasion ability of osteosarcoma cells (143B), which were positively correlated with the dose and cytotoxicity to tumor cells (Figure 2C). The *in situ* osteosarcoma model also demonstrated the remarkable anti-tumor effect of the drug-loaded nanoparticles. The tumor mass in the HA-BSA-PTX treatment group was significantly lighter than that in the control group, and the survival time was considerably extended (Figure 2E). Additionally, the research findings in Figure 2D suggested that the material could induce the upregulation of ALP and OCN expression, promote calcium ion mineralization deposition, and effectively repair bone defects. The drug delivery system not only fulfilled the requirements as a drug carrier, but also had a bone regenerative effect. With its potential for drug sustained release, anti-tumor and osteogenesis, it holds promising prospects as an adjunct treatment for osteosarcoma.

Integrating a targeted ligand into the structure of an anti-tumor drug delivery system can improve the therapeutic efficiency of anti-bone tumor drugs by creating a tumor-targeted drug delivery system (TTDS) (Nicolas et al., 2013). Ideally, anti-bone tumor drugs would selectively accumulate at the target site, resulting in a high local concentration, while the concentration in other non-disease sites should be kept low to prevent adverse effects. For instance, Sun et al. (2018) synthesized nHAp functionalized with FA and loaded DOX into FA-conjugated nHAp (i.e.,DOX@HAP-FA). FR is expressed at low levels in normal cells but is upregulated in many human tumors, making it a potential target for drug-targeted therapy. The nHAp carrier, with a diameter of approximately 100 nm, accumulates at the tumor site through the permeability and retention effect (EPR), while the FA ligand promotes cellular uptake of nHAp through receptor-mediated endocytosis. In simulated acidic microenvironments of tumor cells, DOX@HAP-FA exhibited a faster drug release rate, with a gradual increase to 74% and 82% at pH 6 and pH 5.5, respectively. Similar results were also found in Ansari et al. (2020) study, which further verified that FA-modified nHAp can target tumor cells overexpressing FR. The *in vitro* cytotoxicity of DOX@HAP-FA to human liver cells (HL-7702) and human breast cancer cells (MCF-7) was much lower than that to FR-overexpressing human liver cancer cells (HepG2). The *in vivo* anti-tumor effect of DOX@HAP-FA was evaluated by tumor volume, and on the 21st day, the DOX@HAP-FA group showed the strongest inhibitory effect on tumor growth compared to the free DOX group. The average tumor volume increase in the DOX@HAP-FA group was only 2.5 times, while in the free DOX group, it was 10 times. The absence of DOX@HAP-FA in the major organs of mice and the lack of significant weight loss indicate that the high targeting specificity to FR significantly reduces the toxic side effects of free DOX. TTDS enhances chemotherapy specificity and efficacy, reducing adverse effects on healthy tissues. This strategy can also target specific receptors expressed in bone tumors for efficient delivery of loaded drugs to bone tumor cells.

The drug delivery system using nHAp as a carrier also has limitations in clinical treatment of bone tumors. It not only needs to kill residual tumor cells, but also requires the ability to fill bone defects and promote bone regeneration. Therefore, the development of local sustained-release scaffold materials has become a crucial research direction for bone tumor treatment. Rong et al. (2016) developed a strategy for the treatment of osteosarcoma by implanting PLGA nanoparticles (ADM-PLGA-NHAC) loaded with ADM into a porous nHAp/collagen scaffold. This approach enables the local release of chemotherapy drugs and promotes bone repair. The drug-loaded scaffold showed sustained drug release, with a drug release rate of about 17.6% within 24 h and reaching 65% within 28 days, indicating that it is suitable for long-term drug release to prevent damage to normal tissues caused by high drug concentrations. *In vitro* anti-tumor experiments demonstrated that ADM-PLGA-NHAC exhibited strong anti-tumor activity against osteosarcoma MG63 cells, which increased over time. The implanted scaffold in mice resulted in significantly smaller tumor volumes compared to the control group.

**FIGURE 3:**
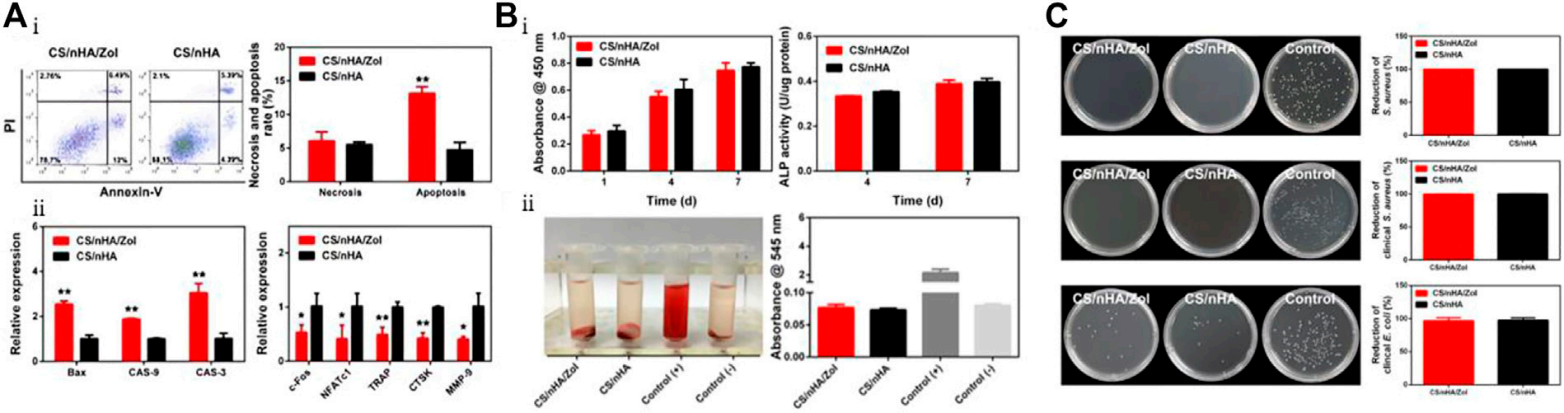
**(A)** Apoptosis and gene expression after co-culturing GCTBs with CS/nHAp/Zol. **(Ai)** Analysis of apoptosis and necrosis in GCTBs cells co-cultured with CS/nHAp for 2 days using flow cytometry. Percentage of necrotic and apoptotic cells in GCTBs. CS/nHAp/Zol upregulated the apoptotic rate of the cells. **(Aii)** Relative expression of apoptotic and osteoclast-related genes in GCTBs after co-culturing with CS/nHAp/Zol for 2 days. **(B)** Biocompatibility and osteogenic differentiation-promoting ability of CS/nHAp/Zol. **(Bi)** Cell proliferation activity of hBMSCs co-cultured with the scaffold for 1, 4, and 7 days as detected by CCK-8, and the effect of co-culturing with the scaffold for 4 and 7 days on hBMSCs ALP activity. **(Bii)** Hemolysis test of the scaffold materials, and both groups of scaffolds did not cause hemolysis. **(C)** Antibacterial ability of CS/nHAp/Zol. From top to bottom are the bacterial counts and antibacterial rates of two types of scaffolds co-cultured with standard *Staphylococcus aureus*, clinical *Staphylococcus aureus*, and clinical *Escherichia coli* for 24 h. Reproduced with permission (Lu et al., 2018b). Copyright 2018, Materials Science and Engineering: **(C)**.

Lu et al. (2018b) developed a composite material composed of CS and nHAp loaded with Zol that has potential applications in supporting fillers, suppressing tumors, providing anti-infection effects, and repairing bone defects. As shown in Figure 3A, the CS/nHAp/Zol material effectively inhibits the proliferation of giant cell tumor of bone (GCTB) cells by upregulating pro-apoptotic genes, increasing the apoptosis rate and necrosis rate of GCTBs to 14% and 7%, respectively—significantly higher than the control group loaded with Zol. Moreover, the material downregulates the expression of osteoclast-related genes, which reduces the bone-resorbing ability of GCTBs. In Figure 3B, the results of the CCK-8 assay and hemolysis test demonstrate that the material exhibits low toxicity to human bone marrow mesenchymal stem cells (hBMSCs). The ALP activity test also shows that Zol does not hinder osteogenic differentiation. Additionally, as shown in Figure 3C, the incorporation of CS into the material demonstrates excellent antibacterial activity with inhibitory rates against clinically pathogenic *Staphylococcus aureus* and *Escherichia coli* of 99.84% ± 0.14% and 99.92% ± 0.14%, respectively.

In a related investigation, Tan W and colleagues incorporated MET into a scaffold made of nHAp (Tan et al., 2021). They developed a dual-functional scaffold (i.e.,PLLA/nHAp/MET) composed of PLLA/nHAp loaded with MET, which has the potential to both kill tumor cells and promote bone regeneration. The PLLA/nHAp/MET scaffold effectively induced apoptosis of osteosarcoma cells (Saos-2) *in vitro*, resulting in an early cell apoptosis rate of 12.87% and a late cell apoptosis death rate of 15.86%, and arrested the cell cycle. Moreover, after 7 days, the osteogenic culture medium containing the PLLA/nHAp/MET scaffold showed higher ALP expression compared to the PLLA/nHAp scaffold, indicating that the addition of MET enhances the osteogenic differentiation of hBMSCs.

In addition to its use in chemotherapy drug delivery, nHAp has also shown promise as a gene transfer vector for gene therapy (Yang et al., 2021). As a non-viral gene carrier, nHAp is easily prepared and avoids the risk of triggering host immune reactions. It can also aggregate and adhere to tumor cell membranes, activate endocytosis, and increase nHAp internalization in the cytoplasm, thus reducing toxicity to normal cells and demonstrating its high safety (Chen et al., 2017; Ke et al., 2020). Moreover, nHAp’s large surface area and easy modification make it advantageous in achieving efficient gene delivery by adjusting its physicochemical properties and functionality (Komuro et al., 2019). Cheang et al. (2018) loaded the HSV-TK gene into GO-nHAp composite materials and combined it with ganciclovir to treat breast cancer. HSV-TK is a commonly used “suicide” gene in tumor gene therapy research. The introduction of GO in the composite material resulted in a significant increase in the specific surface area of nHAp from 17.25 m^2^/g to 91.85 m^2^/g, creating more binding sites for HSV-TK. Upon phosphorylation of HSV-TK, the prodrug ganciclovir (GCV) was activated, which was converted into a potent cytotoxic drug that triggered DNA damage response, induced apoptosis in breast cancer cells, and decreased the viability of human breast cancer cell lines MDA-MB-231 and MCF7 by nearly 70%, while increasing the apoptosis rate by about 22%. In contrast, the viability of the human normal breast cell line MCF 10A remained at 90%, with an apoptosis rate of only 9.3%. These results demonstrate that GO-nHAP complexes can be a promising gene delivery vehicle for anti-tumor therapy, effectively inhibiting cancer cell proliferation and inducing apoptosis, while being well-tolerated by normal breast cancer cells.

Despite its potential in gene delivery, nHAp has some limitations, such as poor dispersibility, particle aggregation, difficult cell internalization, and potential to trigger immune reactions *in vivo*. To address these issues, Xu et al. (2015) modified nHAp by increasing its surface positive potential through polyethylene imine (PEI) coating. The resulting modified nHAp (MnHAP) exhibited a particle size of less than 200 nm and a surface zeta potential of +35.4 MV, which prevented particle aggregation, facilitated adhesion and internalization on the surface of tumor cells, and enabled binding of more siRNA with negative charges. The transfection efficiency of MnHAP/fluorescent FAM-labeled siRNA complexes was significantly higher than that of siRNA alone, and comparable to that of the commercial transfection reagent Lipofectamine^®^2000. Moreover, MnHAP exhibited lower cytotoxicity than the marketable transfection reagent when tested against the human liver cancer cell line BEL-7402. However, despite the fact that nHAp can already be used as a gene transfer vector for delivering target genes to tumor cells, there have been few cases of its application in bone tumor therapy, which highlights the need for further research in this area.

### 3.2 Application of nHAp in PTT or MFH

In addition to conventional radiotherapy, there are other options available to address recurrence and bone defects associated with postoperative bone tumors. One such strategy is to combine HAp with photothermal therapy (PTT) or magnetic fluid hyperthermia (MFH) for *in situ* ablation of tumor cells. Table 2 presents some functional materials based on nHAp for PTT/MFH.

**TABLE 2:** Composition and synthesis methods of various nHAp composites for PTT and MFH and their effects on tumor tissue.

Material	Method of synthesis	Tumor therapy method	Effect	Ref
nHAp、CS、GO	Hydrothermal;Freeze Drying	PTT	This material exhibited an excellent photothermal response under 808 nm NIR irradiation, efficiently ablating HOS, and promoting hBMSC osteogenic differentiation and tissue regeneration through the BMP2/Smad pathway	Ma et al. (2020)
nHAp、Gr	3D printing	PTT	Photothermal treatment promotes the proliferative activity of MC3T3-E1 cells with excellent efficiency, which can accelerate bone regeneration	Zhang and Ma (2021)
nHAp 、CS、CD	Freeze Drying	PTT	The CD-doped scaffold effectively promoted adhesion and osteogenic differentiation of hBMSCs, while significantly inhibiting the proliferation of UMR-106 osteosarcoma cells under NIR irradiation. Additionally, the scaffold demonstrated enhanced antibacterial activity	Lu et al. (2018a)
nHAp、PLGA、Fe_3_O_4_	Freeze Drying	MFH	The material’s surface possesses high porosity and excellent mechanical properties. When exposed to an external magnetic field, it generates high temperatures that selectively ablate tumor cells with minimal impact on normal tissues. Furthermore, the material promotes bone formation *in vivo* by accelerating the process	Li et al. (2019)

Abbreviations: CD, Zero-dimensional carbon dots; Gr, graphene nanoplatelets; hBMCs, human bone marrow stromal cells; NIR, near-infrared laser.

Unlike nHAp materials used in DDS, photothermal functionalized materials use photothermal agents that accumulate at the tumor site to provide local high temperatures under NIR irradiation. This kills residual tumor cells and opens up a promising pathway for precise cancer treatment (Dang et al., 2018; Liu et al., 2018).PTT has emerged as a promising and cost-effective cancer treatment, and various types of photothermal agents, including gold nanomaterials, carbon nanomaterials, polydopamine, and black phosphorus, have been incorporated into scaffolds for this purpose (Yang et al., 2018; Liao et al., 2021; Yao et al., 2021). Among these agents, graphene-based materials have gained attention due to their high photothermal conversion efficiency, biocompatibility, and low cytotoxicity (Ma et al., 2020; Shuai et al., 2022).

**FIGURE 4:**
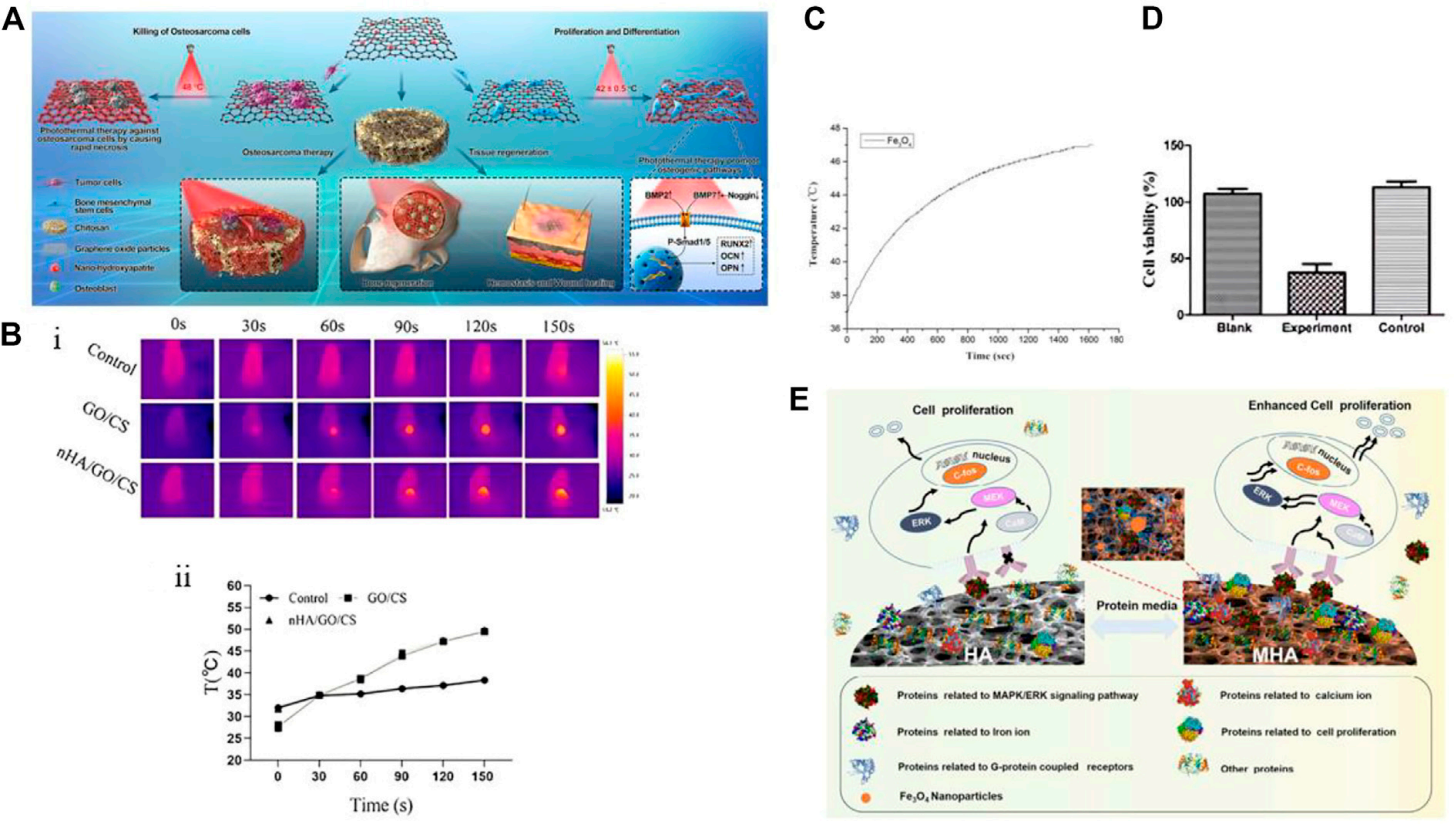
**(A)** Schematic diagram of the preparation and biological application of nHAp/GO particles and nHAp/GO/CS scaffolds. **(B)** Photothermal performance of nHAp/GO/CS scaffold on osteosarcoma after irradiation with near-infrared (NIR) at 808 nm (0.6 W/cm^2^) for 150 s. *In vivo* infrared thermal image **(Bi)** and temperature rise curve **(Bii)** of HOS tumor tissue. **(C)** Magnetic heating performance of PLGA/MF-nHAP under a frequency of 500 kHz and a magnetic field intensity of 3 mT. The material temperature increased with increasing reaction time. **(D)** Cell survival rate of MG-63 cells co-cultured with nanoparticles and exposed to a magnetic field for 30 min, measured by CCK-8 assay. The experimental group had PLGA/MF-nHAP and an external magnetic field applied, the Black group had no nanoparticles, and the Control group had PLGA/MF-nHAP but no external magnetic field applied. About 78% of MG-63 cells in the experimental group were non-viable after magnetic heating treatment. **(E)** Mechanism of MHAp scaffold material in promoting the proliferation of MC3T3-E1 cells. **(A)**, (Bi) and (Bii) Reproduced with permission (Ma et al., 2020). Copyright 2020, Materials Today. **(C)**, **(D)** Reproduced with permission (Li et al., 2019). Copyright 2019, Regenerative Biomaterials. **(E)** Reproduced with permission (Zhu et al., 2017a). Copyright 2017, ACS Nano.

In a study by Ma et al. (2020), a temperature-controlled multifunctional nHAp/graphene oxide/chitosan scaffold material (i.e., nHAp/GO/CS) was designed and constructed, as shown in Figure 4A. Upon NIR irradiation at 808 nm (0.6 W/cm^2^), the GO-coated scaffold could heat up the osteosarcoma site to 49.9°C after 150 s and stabilize at 48°C, while the temperature of the surrounding tissue was much lower than that of the tumor tissue (mean temperature 38.5°C ± 0.6°C), thus reducing damage to normal cells (Figure 4B). In addition to ablating tumor cells, the heat generated by photothermal therapy (PTT) can also accelerate bone regeneration. The scaffold synergistically promotes hBMSC osteogenesis with nHAp under conditions of 42°C ± 0.5°C, and after being implanted into the rat skull defect area for 8 weeks, the bone volume per tissue volume (BV/TV) of the scaffold can reach 20.36%. *In vitro* studies indicate that NIR radiation and nHAp may promote osteogenesis by inducing BMP upregulation and Noggin downregulation, activating downstream genes Smad1 and Smad5 phosphorylation, further upregulating osteogenic genes, and improving osteogenic differentiation. Researchers have been exploring the use of thermal stimulation to accelerate osteogenesis since as early as 2012, and the findings have shown that mild thermal stimulation can aid in early osteogenic differentiation (Chen et al., 2012). Moreover, research results indicate that mild thermal stimulation not only promotes early osteogenic differentiation but also dynamically affects the expression of osteogenic genes, such as BMP2, Runx2, and OPN(Li et al., 2018; Ma et al., 2020).

MFH relies on exposing magnetic nanoparticles (MNPs) to high-frequency alternating magnetic fields (AMF) to convert electromagnetic energy into heat energy, which achieves targeted heating from the inside out to avoid damage to surrounding normal tissues (Kaur et al., 2016). Currently, most MNPs used are Fe_3_O_4_ particles with a diameter between 10 and 20 nm, which generate higher heat under smaller magnetic fields, improving the efficiency of tumor ablation (Kawashita et al., 2005; Giouroudi and Kosel, 2010; Jeyadevan, 2010; Mondal et al., 2017a; Niu et al., 2017). Li et al. (2019) prepared multifunctional magnetic scaffold materials (i.e., PLGA/MF-nHAP) using Fe_3_O_4_ nanoparticles, nHAp, and PLGA through a freeze-drying method to study their anti-tumor and bone repair capabilities. The Fe_3_O_4_ nanoparticles produced had a diameter of only 5 nm, and the nHAp particle size was 50 nm, which is much smaller than the critical particle size of superparamagnetic nanoparticles. In Figures 4C, D, the temperature of Fe_3_O_4_ particles exhibited a positive correlation with reaction time and eventually stabilized at 47°C under the condition of 500 kHz frequency and 3 mT magnetic field intensity. This temperature is higher than the highest tolerable temperature of 43°C for human osteosarcoma cells (MG-63) within the range of normal cell tolerance (Habash et al., 2006). Similar to previous research results, the presence of Fe_3_O_4_ nanoparticles can promote the adhesion, proliferation, and differentiation of hBMCs, regardless of the presence of a magnetic field. However, this effect is significantly enhanced under the induction of an external static magnetic field (SMF) (Tran and Webster, 2011; Zhu et al., 2017a). The introduction of Fe_3_O_4_ nanoparticles has also improved the microstructure of the scaffold by creating a porous surface that promotes molecular interactions with cells and increasing the compressive strength of the material to meet the mechanical performance requirements of implant materials. Although the PLGA/MF-nHAP scaffold has been shown to have good osteogenic properties *in vivo* through tissue staining and micro-CT images, there are no *in vivo* anti-tumor results to evaluate the MFH effect of the scaffold.

Magnetic nanoparticles (MNPs) can be used for tumor treatment by converting electromagnetic energy into heat energy through Néel-Brownian relaxation when exposed to high-frequency AMF. However, they also have good bone-inducing properties when used in SMF(Rosensweig, 2002; Shuai et al., 2018). To understand the mechanism by which magnetic materials promote cell proliferation, Zhu et al. (2017a) investigated how magnetic materials promote cell proliferation (Figure 4E). The altered interaction between proteins and magnetic nanoparticles on MHAp stimulated by an external magnetic field (known as “protein corona”) can enhance cell proliferation. The magnetic field can induce changes in the conformation of adsorbed proteins, leading to changes in protein activity and exposure of certain domains that can interact with cell-surface receptors, leading to increased cell signaling and proliferation. The study suggests that the application of an external magnetic field to MHAp could be a potential method to enhance its efficacy for promoting cell proliferation and tissue regeneration. The study on the protein corona of magnetic hydroxyapatite (MHAp) found that the composition and formation of the protein corona can affect the adhesion, migration, proliferation, and differentiation of cells. The study also found that MHAp tended to adsorb proteins associated with cell proliferation, particularly those that act as upstream promoters for the MAPK/ERK signaling pathway. These findings provide important insights into the molecular mechanisms of cell proliferation on magnetic scaffolds and can guide the design of bifunctional magnetic materials for anti-tumor actions and bone-defect repair after bone-tumor surgery.

PTT and MFH are emerging therapeutic methods that offer advantages such as low toxicity, reduced damage to normal tissues, ease of operation, and controllability. However, their application is limited. Photothermally functionalized scaffolds mostly rely on the first NIR window (NIR-I, 650–950 nm) laser wavelength, which has low tissue penetration and higher scattering compared to the second NIR window (NIR-II, 1,000–1,700 nm) (He et al., 2018; Du et al., 2021). This makes it difficult for NIR-I responsive photothermal functionalized scaffolds to effectively eliminate deep bone tumor cells. Increasing the power of the NIR-I laser (>0.33 W/cm^2^) for a longer period of time can cause damage to superficial tissues (Ding et al., 2014; Xu et al., 2022). In the case of MFH, the efficiency of magnetic energy conversion to heat energy depends on the size of the magnetic particles, with nanoparticles being far superior to microparticles. This size requirement poses a challenge in the preparation process, and providing and controlling the required strength of the alternating magnetic field also increases the difficulty of MFH(Kozissnik et al., 2013; Li et al., 2019).

### 3.3 Application of nHAp doped with trace elements in the diagnosis and treatment of bone tumors

The structural formula of nHAp is Ca_10_(PO_4_)_6_(OH)_2_. The anionic groups (PO_4_^3-^, OH^−^) and cationic group (Ca^2+^) can be replaced by other ions, offering the possibility to incorporate other elements (Šupová, 2015; Ratnayake et al., 2017). Some of these elements also have interesting roles in the treatment of bone tumors, as shown in Table 3.

**TABLE 3:** Ionic exchange/doping in the nHAp structure, useful for bone tumor therapy and diagnostics.

Substitution	Effect	Ref
^89^Sr	The radioisotopes^89^Sr and^32^P emit β radiation and are effective treatments for bone tumors, as they not only target the tumor cells but also promote bone regeneration in the irradiated area	de Rezende et al. (2019)
Se	Excessive generation and accumulation of ROS can activate the JNK pathway, inhibit the Akt/mTOR pathway, and induce tumor cell autophagy and caspase-dependent endogenous apoptosis	Wang et al. (2015b), Zhou et al. (2019a), Li et al. (2020)
Eu	The material induces apoptosis of osteosarcoma cell lines U-2 OS, Saos-2, and MG-63, while not affecting the proliferation activity of HuASCs. Furthermore, the material possesses bioimaging functionality	Sikora et al. (2019), Faria et al. (2021)
Fe	The presence of Fe^3+^ gives the material superparamagnetic properties. This makes it an effective contrast agent for improving the accuracy of MRI scans, detecting cancer growth, and facilitating targeted thermal energy under AMF. Additionally, it can kill tumor cells, promote osteogenesis and vascular differentiation in SMF, and even aid in repairing bone defects	Zhu et al. (2017a), Adamiano et al. (2018), Ereath Beeran et al. (2019), Li et al. (2019)
Gd	The addition of nHA endows the material with PL, which enables *in vivo* imaging	Chen et al. (2011), Zhang et al. (2020)

Abbreviations: HuASCs, human adipose tissue-derived stromal cells; PL, photoluminescent functionality.

**FIGURE 5:**
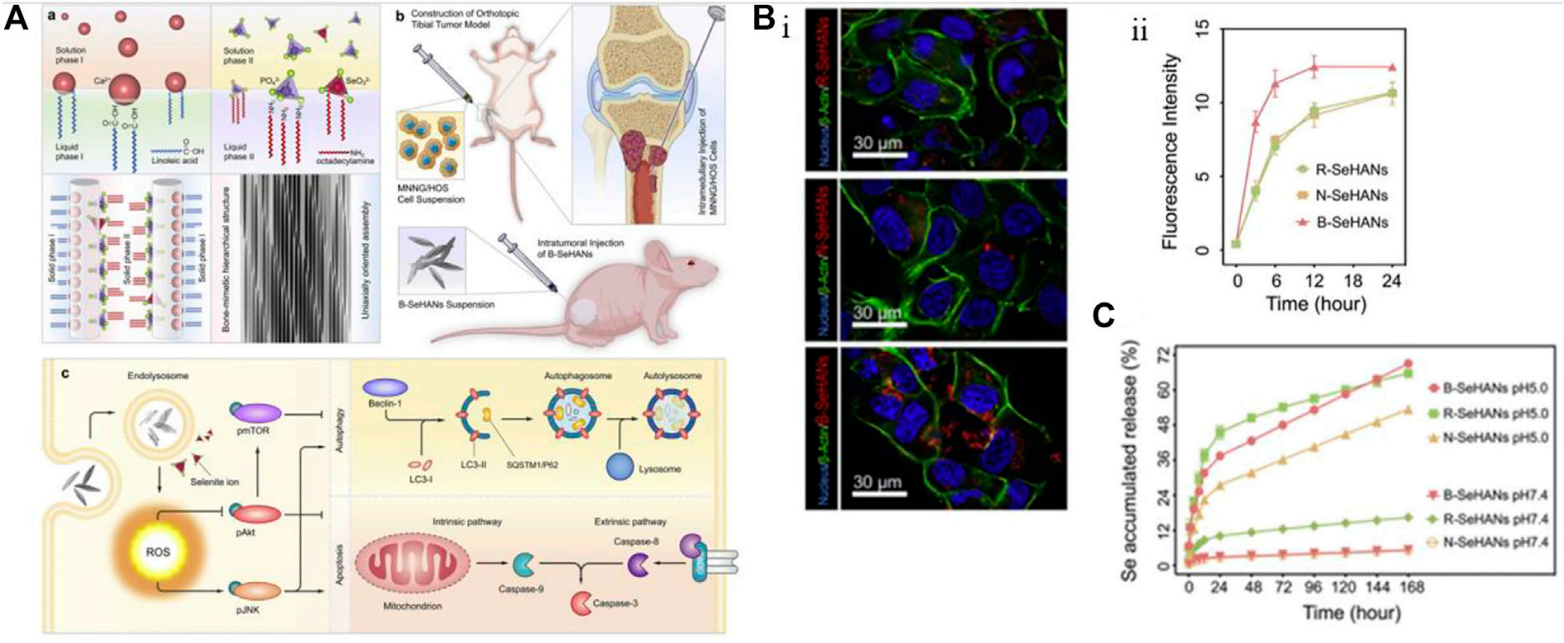
Incorporating selenium element into nHAp material to achieve anti-tumor effects. **(A)** Synthesis method and schematic diagram of anti-osteosarcoma effect of B-SeHANs. **(B)** The uptake results of B-SeHANs, R-SeHANs, and N-SeHANs by MNNG/HOS cells. After co-cultured for 12 h, the LCSM images of the three SeHANs internalized by MNNG/HOS cells **(Bi)**, and the SeHAN uptake by MNNG/HOS cells was quantified by FCM and plotted as a curve **(Bii)**. Compared with non-biomimetic SeHANs, B-SeHANs accumulated more in cells. **(C)** Compared with R-SeHANs and N-SeHANs, the release kinetics of selenium ions from B-SeHANs showed better acidic reaction characteristics. Reproduced with permission (Li et al., 2020). Copyright 2018, J Biomed Nanotechnol.

Using nHAp as a carrier, trace elements with anti-tumor functions can be doped to achieve targeted therapy due to the high uptake efficiency of tumor cells for nHAp. Selenium (Se) is a trace element that has been shown to help prevent bone, breast, prostate gland, lung, and liver tumors. It can also inhibit the proliferation and migration of tumor cells while protecting healthy tissue (Wang et al., 2016). Li et al. (2020) designed and prepared a biomimetic mineralized selenium-doped nHAp material (i.e., B-SeHANs). As shown in Figure 5A, selenite partially replaces phosphate, guiding the uniaxial oriented crystal growth to simulate the hierarchical structure of natural bone. Figures 5B, C show that the biomimetic layered structure of B-SeHANs enhances cell uptake and intracellular degradation, with the highest release rate of Se in an acidic environment. *In vitro* evaluation results demonstrate that compared to two non-biomimetic SeHANs, namely, rod-like SeHANs (i.e.,R-SeHANs) and needle-like SeHANs (i.e., N-SeHANs), B-SeHANs exhibit high intracellular degradation ability and anti-tumor function. After being treated with 20 μg/mL B-SeHANs for 24 h, the average apoptosis rate of human MNNG/HOS osteosarcoma cells was approximately 36%, and the tumor activity decreased to about 50% after 48 h. *In vivo* evaluation of the tibial osteosarcoma model further confirmed the anti-tumor ability of B-SeHANs and its ability to reduce tumor-induced bone destruction. The researchers also inferred the mechanism by which B-SeHANs kill osteosarcoma cells by demonstrating that the activation of the ROS-mediated JNK pathway and the inhibition of the Akt/mTOR pathway induce subsequent autophagy and caspase-dependent cell apoptosis. This material can have a dual role in the postoperative treatment of bone tumors by combining the unique functions of Se with the promotion of bone repair by nHAp.

In addition to the material structure, the amount of Se substitution in nHAp also affects the toxicity of bone tumor cells. Prepared three different levels of selenium-substituted nHAp: HAp, 3%Se-HAp, and 10%Se-HAp, by doping selenite ions at Se/P mass ratios of 0%, 3%, and 10%, respectively. By comparing the selenite release curves of the three types of selenium-substituted nHAp, it was found that the release amount of 3%Se-HAp was higher than that of 10%Se-HAp (89.72% and 30.64%, respectively). The anti-tumor activity of HAp, 3%Se-HAp, and 10%Se-HAp was tested in an *In situ* nude mouse osteosarcoma model constructed by injecting SOSP-9607. After 30 days of injection of the materials into the osteosarcoma, the 3%Se-HAp group exhibited the greatest delay in tumor volume growth, and had the least residual amount on the tumor surface, indicating better biodegradability, selenite ion release, and tumor growth inhibition.

On the other hand, platinum (Pt) is a widely recognized component of highly effective anti-cancer drugs such as cisplatin, carboplatin, and oxaliplatin. In clinical settings, Se compounds have been combined with Pt-based drugs to specifically alleviate cisplatin-related nephrotoxicity and bone marrow suppression in cancer patients (Hu et al., 1997; Ghorbani et al., 2013). This noteworthy ability of Se is thought to arise from the formation of intermediate Se–Pt complexes that exhibit reduced Pt toxicity without compromising the therapeutic efficacy of cisplatin (Zhang et al., 2008; Liu et al., 2010). A recent study conducted by Barbanente A et al. investigated the use of selenite-doped hydroxyapatite nanoparticles loaded with a hydroxyapatite-binding anti-tumor platinum complex (i.e., PtPP–HASe) to selectively reduce cancer cell proliferation without affecting bone marrow stem cells. The release kinetics of Pt and Se were evaluated and a pH-dependent release was observed for Se, but not for the drug. Moreover, the percentage of Se substitution affected the release of Pt. After 7 days, the cumulative release of Se and Pt was ∼10 and ∼66 wt%, respectively. At a Pt/Se ratio of 8, the released Pt and Se species selectively reduced the cell numbers of human prostate (PC3) and human breast cancer cells (MDA-MB-231). These findings suggest a new strategy for treating bone tumors, which involves co-administering Se with chemotherapy drugs to synergistically improve efficacy and overcome the drawbacks of traditional chemotherapy.

Besides Se, other elements have been explored for the treatment of bone tumors. For instance, Kalniņa D et al. prepared nanocrystalline vanadium-doped HAp by substituting some phosphate with vanadate (Kalniņa et al., 2019). The results showed that V(v)-doped nanocrystalline HAp had cytotoxic effects against human chondrosarcoma cells SW1353. However, further *in vivo* data are needed to confirm its efficacy. Similarly, Sikora M et al. used a hydrothermal method to introduce europium (III) ions (Eu^3+^) into nHAp/PLLA material and tested its cytotoxicity on three human osteosarcoma cell lines (U-2 OS, Saos-2, and MG-63) and HuASCs (Sikora et al., 2019). The results demonstrated that the material had high cytotoxicity against all OSA cell lines, but no significant metabolic effects were observed in MG-63. This may be attributed to the differences in gene expression patterns and high heterogeneity among osteosarcoma cell lines. Furthermore, the material did not affect the vitality and proliferation rate of HuASCs, indicating its potential bone immunomodulatory properties and ability to promote osteogenic characteristics for HuASCs. Additionally, introducing Eu^3+^ into the nHAp lattice allowed for imaging function, thereby expanding the application of nHAp in tumor imaging diagnosis. Zhou R et al. utilized hyaluronic acid (HA) as a template for the co-precipitation synthesis of Eu/Ba co-doped nHAp (Zhou et al., 2020). The resulting co-doped nHAp exhibited strong red fluorescence under ultraviolet light (365 nm), as evidenced by the excitation spectrum of HA@nHAP: Eu/Ba. This co-doped nHAp had high doping efficiency, resulting in a better signal for X-ray fluorescence computed tomography. Additionally, low molecular weight HA (<10 kDa) serves as a receptor for CD44, which is overexpressed on the tumor cell membrane. Therefore, HA@nHAP: Eu/Ba exhibits tumor-targeting properties and can be utilized as a probe for targeted tumor cell imaging.

In the last few decades, several nHAp materials have been developed as contrast agents for cancer imaging. Among them, nHAp doped with gadolinium (Gd^3+^) has been used as a tracer for magnetic resonance imaging (MRI) to enhance the paramagnetic longitudinal relaxation of MRI. Besides Gd-based agents, there are also Fe-based agents used for MRI, including maghemite (γ-Fe_2_O_3_), hematite (α-Fe_2_O_3_), and magnetite (Fe_3_O_4_) (Bharath et al., 2014). These iron oxide nanoparticles are often referred to as surface-engineered superparamagnetic iron oxide nanoparticles (SPIONs) (Iafisco et al., 2016). While these agents have potential for cancer treatment, they have limitations such as nanoparticle oxidation and self-aggregation due to their high surface energy, leading to segregation by the reticuloendothelial system (RES) and a sudden temperature rise when an alternating magnetic field (AMF) is applied (Mondal et al., 2017b; Ereath Beeran et al., 2019). However, these problems can be overcome by incorporating these magnetic compounds into inorganic materials such as HAp (Abbasi Aval et al., 2016). Furthermore, SPIONs as contrast agents are often affected by the accumulation of Fe ions in soft tissues, and Fe-doped nHAp seems to be a better choice, which can produce higher MRI contrast (Mondal et al., 2017a; Adamiano et al., 2018). Ereath Beeran A et al. investigated the potential of iron oxide-embedded nHAp for controlled hyperthermia treatment and MRI contrast efficiency *in vitro*. They tested concentrations ranging from 0.05 mM to 0.25 mM of the nanoparticles on HeLa cells and found a significant reduction in signal intensity of T2 weighted MRI with increasing concentrations (Ereath Beeran et al., 2019). The fluctuation in relaxation induced by the nanoparticles, based on proton relaxation in reference to the local magnetic field and occurring on spin-lattice relaxation (T1) and spin-spin relaxation (T2), was the cause of the results. These findings suggest that magnetic nanoparticles embedded in nHAp have potential as negative contrast enhancers in MRI.

nHAp can also be doped with radioactive isotopes such as Strontium-89 (^89^Sr), Phosphorus-32 (^32^P), and Gadolinium-159 (^159^Gd) (de Rezende et al., 2019; Liu et al., 2019; Kargozar et al., 2022). These isotopes can serve as internal radiation sources that emit β and γ radiation, which can effectively kill tumors while also providing imaging functions. Additionally, these isotopes can be dissociated from chelating agents to prevent their deposition in normal tissues, reducing the risk of toxicity. By combining internal radiation therapy and imaging, these radioactive nHAp materials have great potential for the diagnosis and treatment of cancers.

When using ion-doped nHAp materials, it is important to be aware of some issues. For instance, Díaz et al. conducted an MTT assay to analyze the effect of PLLA scaffolds containing Fe-doped hydroxyapatite (i.e., FeHAp) particles on the metabolic activity of MC3T3-E1 cells (Díaz et al., 2018). They observed cytotoxicity for FeHAp content above 20%. While the addition of trace elements can help optimize performance and expand the functions of nHAp, it is important to pay attention to the doping concentration of ions. Neglecting this point may reduce the biocompatibility of the material and disrupt the dynamic balance in the human body.

### 3.4 Application of nHAp in anti-infection therapy after bone tumor surgery

After surgical treatment, such as the removal of malignant tumors in bones, there is a risk of infection in the wound. The most important pathogens that cause infections in bones and joints and severe sepsis are *Staphylococcus aureus* and *Pseudomonas aeruginosa*. If bacteria adhere to the implant surface, they tend to induce the production of biofilm, and the bacteria develop resistance rapidly after biofilm formation (Zhou et al., 2012; Yu et al., 2014). It’s crucial to administer follow-up treatment that ensures the local maintenance of effective antibiotic concentrations in the wound for effective bone infection treatment and prevention. Long-term intravenous administration of high doses of antibiotics may harm vital organs, and sublethal drug concentrations and poor tissue circulation postoperatively can lead to bacterial resistance (Zimmerli, 2014; Inzana et al., 2016). The use of HAp composites with anti-infective properties can help to solve these problems by providing local antimicrobial effects and reducing the risk of serious systemic complications. Additionally, HAp can aid in filling and integrating reconstruction at the site of bone defects.

**FIGURE 6:**
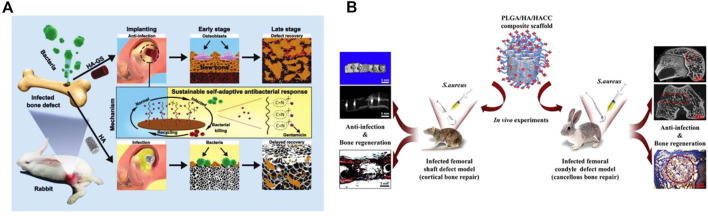
**(A)** Schematic diagram of stimulus-responsive HAp-GS material fabrication and its biological applications. **(B)** Schematic diagram of the experimental protocol for PLGA/HAp/HACC antimicrobial materials. **(A)** Reproduced with permission (Jin et al., 2019). Copyright 2019, Advanced Functional Materials. **(B)** Reproduced with permission. Copyright 2018, Acta Biomater.

Several nHAp composites loaded with antibiotic have been shown to have significant antimicrobial activity. Zhou et al. (2012) developed an nHAp/β-TCP scaffold coated with cationic liposome-coated cefotaxime (CLCs), which was effective in inhibiting the formation of *S. aureus* biofilm by locally releasing antibiotics and maintaining an effective concentration. However, drug release studies of the loaded scaffold revealed a burst release phenomenon before the release of CLCs, resulting in a short-term high local drug concentration that may affect the proliferation and differentiation of osteoblasts. Previous research has demonstrated that adjusting the types and doping ratios of components in composite materials can modulate the controlled release rate of loaded drugs. In comparison to single-phase nHAp material loaded with enrofloxacin, the enrofloxacin/sodium alginate/nHAp composite material prepared *via* the wet chemical method can gradually degrade in the body. By comparing the drug release curves of the two materials over a 30-day period, it is evident that the addition of sodium alginate results in a gentler drug release curve, with only 80% of the drug being released. This leads to stable and slow drug release, effectively prolonging the release time of enrofloxacin (Venkatasubbu et al., 2011). Therefore, multi-phase composite materials can be selectively designed to maintain drug stability and release by selecting various components while ensuring antibacterial properties. For instance, Jin et al. designed a responsive porous antibacterial implant material for preventing and treating infected bone defects (Figure 6A) (Jin et al., 2019). They used low cytotoxicity ethylenediamine (ED) functionalized polyglycerol methacrylate (PGMA) (PGED) and acid-responsive Schiff base to connect loaded gentamicin sulfate (GS). The release of GS can be triggered effectively in the acidic environment of bacterial metabolism, achieving sustained and responsive targeted antibacterial activity. The material demonstrated high antibacterial performance and excellent bone regeneration ability through *in vitro* antibacterial performance testing and treatment of the infectious rabbit bone defect model.

The ions of certain heavy metals possess bactericidal properties. Doping HAp with these metals can increase the material’s ductility and capacity to withstand shear stress (Ratnayake et al., 2017). Common metals with antimicrobial activity used for HAp doping include silver, zinc, and copper (Kim et al., 1998; Ohtsu et al., 2018; Simon et al., 2019).Silver ions (Ag^+^) can damage the outer membrane of bacteria by disrupting cytosolic proteins. They also inhibit the electron-transport chain of cells by binding to the sulfhydryl groups of metabolic enzymes, and can bind to bacterial DNA and RNA to inhibit bacterial replication, resulting in sterilization effects (Rai et al., 2009; Devasconcellos et al., 2012; Kakinuma et al., 2015). Ag^+^ are commonly used to coat the surface of materials due to their broad-spectrum antibacterial properties and low potential for resistance from bacteria. However, directly coating a material surface with Ag^+^ can result in sudden release of Ag^+^ at an early stage, which may not lead to a lasting antibacterial effect. Therefore, the slow release of Ag^+^ is crucial to achieving lasting antibacterial effects in materials (Liu et al., 2020). Saravanan et al. (2011) utilized the freeze-drying method to prepare a CS/nHAp/Ag^+^ composite material, which was then subjected to *in vitro* antibacterial experiments against *S. aureus* and *Escherichia coli*. The results demonstrated a significant antibacterial effect in the Ag^+^ loaded group. Moreover, subsequent investigations revealed that the experimental group material was capable of controlling the release of Ag^+^ in a manner that roughly matched the growth of new bone tissue. Although Ag^+^ has excellent antibacterial properties, it can be toxic to human cells if its concentration exceeds a certain threshold. To address this issue, it can be doped with minor chemical elements or bioactive compounds for multiphase compounding with nHAp material. This approach is effective in mitigating silver toxicity while maintaining its optimal antimicrobial properties. Geng et al. (2016) doped Ag^+^ and Sr^2+^ ions into nHAp material using a hydrothermal method and immobilized the resulting coating on titanium metal. The addition of Sr^2+^ improved the adhesion, proliferation, and differentiation of osteoblasts on the coating, while reducing the toxicity of Ag^+^ to osteoblasts. The co-doped nHAp coating demonstrated excellent biocompatibility and antibacterial activity, and underwent uniform deposition on titanium, enhancing its hydrophilicity, bioactivity, and nanoscale surface roughness. This coating induced bone regeneration or adhered directly to bone in the early stages after implantation and has significant potential for modifying orthopedic metal implants. However, the dose ratios between elements must be carefully adjusted to achieve a good balance of excellent antibacterial properties and good bioactivity. Further research is needed to better understand the *in vivo* mechanisms of action of metal ion-loaded composites and to avoid serious toxic side-effects from inappropriate application of heavy-metal ions.

In the study mentioned above, Lu et al. (2018b) demonstrated that compounding chitosan with scaffold materials can confer anti-infection properties to the material, suggesting that in addition to loading antibacterial drugs and elements, desired properties can also be achieved through material compounding. CS, which is a deacetylated derivative of chitin, is one such material. CS is a linear polysaccharide composed of glucosamine and N-acetylglucosamine through a β,1-4 glycosidic bond. Due to its structural and compositional similarity to the glycosaminoglycans present in articular cartilage, CS exhibits high biocompatibility and low immunogenicity (Balagangadharan et al., 2017). Furthermore, CS possesses excellent biodegradable, antibacterial, and hemostatic properties (Zhang et al., 2014). HACC, or 2-hydroxypropyltrimethyl ammonium chloride chitosan, is derived from chitosan and has potent antimicrobial properties due to its ability to electrostatically bind to bacterial membranes. By varying the degree of substitution of quaternary ammonium salts, the cytocompatibility and antimicrobial activity of HACC can be optimized (Crismaru et al., 2011; Yang et al., 2016; Yang et al., 2017). Yang et al. 2018 utilized 3D printing technology to produce a PLGA/HAp/HACC copolymer scaffold, where HACC is a quaternary chitosan covalently grafted onto the scaffold (Yang et al., 2018). As shown in Figure 6B, two models of bone defects infected with *S. aureus* were created to evaluate the scaffold’s anti-infection, bone-regeneration, and *in vivo* degradation properties. Results showed that in both the rat femoral stem defect model and the rabbit femoral condyle defect model, the composite scaffold group exhibited no visible signs of bone infection on the surface. Moreover, the scaffold was able to integrate well with the bone tissue, promote significant bone defect healing, and degrade slowly *in vivo*. Addition of HAp could: i) optimize the mechanical properties of the composite scaffold between cancellous bone and cortical bone; ii) slow-down PLGA degradation *in vitro*. The addition of HAp to the PLGA-based polymer scaffold not only helped to prevent dislocation and deformation of the implanted scaffold, but also neutralized the decrease in pH during degradation, preventing a micro-acidic environment from promoting bacterial proliferation. However, it is essential for the scaffold’s degradation rate to match the rate of osteogenesis to achieve successful bone defect repair. Therefore, it is important to investigate whether the degradation rate of the composite material interferes with bone-tissue regeneration. Additionally, while the antimicrobial effect of the composite against Gram-negative bacteria-associated bone infections is promising, further investigation is needed to fully assess its efficacy. The potential impact of combining the composite with antibiotics to improve its anti-infection properties on bone repair is also unknown. If successful, this could improve the prospects for repairing infected cortical and cancellous bone defects.

## 4 Conclusion and outlook

In recent decades, traditional treatment options for primary and secondary bone tumors have shown limited improvement and mainly rely on chemotherapy, radiotherapy, and surgical intervention. These options have several drawbacks, including multidrug resistance, tumor recurrence, severe systemic toxicity, and large bone defects formation, which limit their application and efficacy. Fortunately, there has been considerable research on the application of nHAp as a bone tissue engineering material in the treatment of bone tumors, such as drug delivery carriers, photothermal functional materials, magnetic nanoparticles, and others. Combining traditional treatment options with adjuvant therapy can form personalized treatment strategies to overcome the limitations of existing therapies and improve treatment efficacy. However, there are still several issues with the constructed nHAp materials that require further exploration.

Firstly, nHAp has been found to inhibit the proliferation of various types of tumor cells through different mechanisms. However, several studies have highlighted that pathologic nHAp generated *in vivo* not only promotes tumor cell proliferation and differentiation but also enhances tumor cell invasiveness (Smith et al., 2012; Choi et al., 2015; Cui et al., 2016; Scimeca et al., 2019). Malignant tumor cells actively produce pathologic nHAp, which first forms in the tumor cells and then released into the extracellular matrix. The highly ischemic and hypoxic microenvironment leads to excessive mineral deposition, forming microcalcifications that produce pathologic nHAp (Wilkinson et al., 2016; O'Grady and Morgan, 2018; Clemenceau et al., 2020; Emami et al., 2020). Pathologic nHAp in tumors deposits similarly to physiological mineralization. Therefore, it is necessary to maintain the balance between enhancers and inhibitors of physiological mineralization (Cox and Morgan, 2013). Specific proteins such as topoisomerase II (Topo II) inhibitors, TGF-β inhibitors, anti-OLC biomarkers, *etc.*, can be interfered with to prevent the growth, development, and metastasis of tumors and inhibit tumor microcalcification (Makhoul et al., 2016; Scimeca et al., 2020; Simatou et al., 2020). However, the molecular mechanisms of nHAp involvement in tumor development remain unclear, and it is necessary to further explore the interaction between the physicochemical properties of nHAp and the tumor microenvironment to better use nHAp in the prevention and treatment of tumor metastasis.

Secondly, patients with bone tumors often face the problem of residual tumor cells and bone defects after surgery. It is necessary to develop multifunctional materials that have both tumor treatment and bone repair functions, and to shift from single therapy to synergistic therapy between two or more therapies, enhancing treatment effects (i.e., “1 + 1>2″) (Fan et al., 2017; Zhang et al., 2022). A stimulus-responsive intelligent nHAp drug carrier was designed based on the microenvironment of bone tumors. Considering the special characteristics of bone tumor cells, nHAp was modified to respond to endogenous stimuli, such as low pH, high reducing level, and abnormally expressed enzymes, and used for targeted drug delivery (Zhou et al., 2018; Zhang et al., 2019; Mi, 2020). This increases toxicity to tumor cells, reduces drug dosage, and has low toxicity or even no toxicity to normal tissues while also promoting bone regeneration. The nHAp stimulus-responsive carrier can also respond to exogenous stimuli such as light, ultrasound, magnetic field, and heat to release drugs on demand. For instance, heat-stable drugs can be loaded into nHAp and drug release triggered by changes in external temperature, combined with PTT/MFH to synergistically kill tumor cells and accelerate bone tissue regeneration (Karimi et al., 2016; Qian et al., 2016; Kralj et al., 2017; Chen and Zhao, 2018). Another strategy is to use nHAp for photodynamic therapy (PDT) or sonodynamic therapy (SDT) by loading photosensitizers/sonosensitizers into nHAp and reacting with oxygen to generate ROS under specific wavelength light/low-intensity ultrasound, inducing apoptosis or necrosis of bone tumor cells (Agostinis et al., 2011; Zhu et al., 2020).

Finally, to achieve optimal biocompatibility, the concentration of ions in the lattice of element-doped nHAp material should be close to physiological levels, and the lowest effective concentration should be explored. Micro-nanostructure design should also be introduced when applying functionally doped elements to achieve the goal of synergistically promoting osteogenic effects. Further research is needed to determine the optimal selection of bioactive substances and the best combination between them, as well as how to achieve the optimal proportion of ions released by implanted materials. Additionally, issues such as immune reactions caused by implants need to be addressed (Šupová, 2015; Ratnayake et al., 2017; He et al., 2019).

As a bone implant material, nHAp composite scaffold materials must achieve an optimal balance between mechanical and biological properties. From a biomimetic perspective, the material should be constructed to avoid conflicts between porosity, mechanical strength, and degradation performance. While ensuring sufficient mechanical strength, the scaffold material should match the required gradient mechanical properties of new bone formation as much as possible. The material surface should be rough and porous, the internal pore size should be suitable and interconnected, and the material degradation rate should be coordinated with the rate of bone formation. This is crucial to prevent the synthesized material from having relatively poor biological activity, which may cause inflammatory reactions in the body and be detrimental to cell survival, adhesion, and distribution (Wei and Ma, 2004; Marques et al., 2014).

Although the challenges mentioned above are significant, it is important to note that they are surmountable through continuous development of new processes, materials, and technologies, as well as the optimal combination of various substances. The long-term benefits of advancing this research field far outweigh the potential risks.
